# Immunostimulatory effect of a composition isolated from white peony root oral liquid in the treatment of radiation-induced esophagitis

**DOI:** 10.3892/etm.2013.1227

**Published:** 2013-07-18

**Authors:** ZHIYU WANG, LI SHEN, JUAN WANG, BAOEN SHAN, LI ZHANG, FUHE LU, XIUJUAN GUO, XING LI

**Affiliations:** 1 Department of Biotherapy, Fourth Hospital of Hebei Medical University, Shijiazhuang, Hebei 050011, P.R. China; 2 Centre of Scientific Research, Fourth Hospital of Hebei Medical University, Shijiazhuang, Hebei 050011, P.R. China; 3 Department of Traditional Chinese Medicine, Fourth Hospital of Hebei Medical University, Shijiazhuang, Hebei 050011, P.R. China; 4 Department of Radiotherapy, Fourth Hospital of Hebei Medical University, Shijiazhuang, Hebei 050011, P.R. China

**Keywords:** composition isolated from white peony root oral liquid, immune repair, radiation-induced esophagitis

## Abstract

The aim of this study was to explore the immune repairing effect of a composition isolated from white peony root oral liquid (cWPROL), a traditional Chinese herbal composition, in the treatment of experimental radiation-induced esophagitis in rats. A total of 128 Wistar rats were randomly divided into eight groups, irradiated with 43 Gy ^60^Co γ-rays to induce esophagitis and treated by different methods. Flow cytometry, hematological analysis and immune nephelometry were used to detect the absolute numbers and percentages of CD3^+^, CD4^+^ and CD8^+^ T lymphocytes, numbers and classification of leukocytes, and the levels of IgG and complement C3 in the peripheral blood of the rats at each experimental time point. Following irradiation, the total number of leukocytes, absolute numbers and percentages of CD3^+^, CD4^+^ and CD8^+^ T lymphocytes, and levels of IgG and complement C3 in the peripheral blood of the rats were decreased. Furthermore, the total numbers of leukocytes, absolute numbers and percentages of CD3^+^, CD4^+^ and CD8^+^ T lymphocytes, and levels of IgG and complement C3 in the peripheral blood were higher in the administered with cWPROL by intra-esophageal perfusion compared with those in the untreated irradiated groups, but lower in the groups treated with a mixture of lidocaine hydrochloride, dexamethasone sodium phosphate and gentamicin sulfate. This study suggested that cWPROL is able to repair the impaired cellular and humoral immunity of rats with radiation-induced esophagitis.

## Introduction

For patients with carcinomas of the head, neck, thorax or other parts of the body who have received radiotherapy, lower absolute numbers of T and B lymphocytes compared with those before radiotherapy and an imbalance in the absolute counts of T lymphocyte subsets may be observed in the peripheral blood, which may result in a decreased immune function and increased incidence of radiation-induced esophagitis (1,2). The duration of the impaired cellular and humoral immunity of patients who have received radiotherapy may be between several months and several years, and this phenomenon has also been further verified in experiments with rats irradiated by ^60^Co γ-rays (3). The duration of the impaired immunity is likely due to the inhibitory effect of radioactive rays on the immune system and may have a certain association with the obstinate, intractable characteristics of radiation-induced esophagitis.

A composition isolated from white peony root oral liquid (cWPROL), whose major components are white peony (*Paeonia suffruticosa)*, *Sophora tonkinensis* and *Bletilla striata*, is a prescription formulation independently developed by our investigators and has a good efficacy for the treatment of radiation-induced esophageal toxicity in previous experiments and in clinic (4), however, its functional mechanism remains unclear. This study analyzed the variations of leukocytes and indicators of cellular and humoral immunity in rats with radiation-induced esophagitis and following various treatments. The results demonstrated that cWPROL may exert its preventive and therapeutic effects on the disease by restoring the immunity system that has been damaged by radioactive radiation.

## Materials and methods

### Animals

A total of 128 adult Wistar rats weighing 180–220 g, half male and half female, were provided by the Animal Department of Hebei Medical University (Shijiazhuang, China). The animal care and experimental protocols complied with the internationally recognized guidelines on animal welfare, Regulations for the administration of affairs concerning experimental animais of the State Science and Technology Commission of the People’s Republic of China and the guidelines for the Care and Use of Laboratory Animals of Hebei Medical University. In addition, this study was approved by the Medical Ethics Committee of The Fourth Hospital of Hebei Medical University.

### Medication

The cWPROL was processed according to the formula and provided by the Pharmacy Department of the Fourth Hospital of Hebei Medical University. The concentration of crude medicinal components contained in the cWPROL was 2.88 g/ml and its relative density was 1.18. The major components of cWPROL are white peony root, *Sophora tonkinensis* and *Bletilla striata*. Its production process was as follows: first, the Sophora tonkinensis (100 g) and *Paeonia suffruticosa* root (300 g) are soaked for 2 hours, the solution was boiled for 1 hour and the liquid was removed. The solids were boiled again with new water for 40 min, the solutions of both steps were mixed, filtered through a signle layer of gauze and concentrated to a relative density of 1.07 (between 70–80°C). The solution was refrigerated (2°C) for 48 hours, and filtered again. Second, *Bletilla striata* (150 g) was soaked for 1 hour, boiled for 45 min, liquids removed, boiled with new water for 30 min and the liquids were mixed. The liquids were filtered with a single layer of gauze and mixed with the filtered liquids of the first step. The solution was concentrated to a relative density of 1.18 (between 70–80°C), refrigerated (2°C) for 12 hours, purified water was added to a final volume of 1,000 ml and the solution was homogenized. The liquids were filtered through a single layer of gauze, sealed in polypropylene plastic bottles and sterilized in an autoclave sterilizer (Chenfeng medical apparatus and instruments manufacturing Corp. Ltd, Jixi, Heilongjiang, China; 105°C, 0.25 MPa) for 30 min to obtain the final cWPROL. The western medicines used were lidocaine hydrochloride injection (2%; Fuda Pharmaceutical Corp. Ltd., Shanghai, China), dexamethasone sodium phosphate injection (5 mg; Taikang Pharmaceutical Corp. Ltd, Hangzhou, Zhejiang, China), gentamicin sulfate injection (40,000 U/ml; Tiancheng Pharmaceutical Corp. Ltd, Cangzhou, Hebei, China) and normal saline (250 ml; Tiancheng Pharmaceutical Corp. Ltd.). All injections were administered orally.

### Immunology

Fluorescein isothiocyanate (FITC)-labeled mouse anti-rat CD3 monoclonal antibodies (clonal code: 1F4) and mouse anti-rat FITC-labeled CD4 and RPE-labeled CD8 double-labeled monoclonal antibodies (CD4 clonal code: W3/25, CD8 clonal code: OX8) were provided by AbD Serotec (Raleigh, NC, USA). Serum immunoglobulin IgG and complement C3 kits were purchased from Sun Biotechnology Company (Shanghai, China).

### Animal models of radiation-induced esophagitis

The rats were placed into a specific fixator made of organic glass and, while conscious, the chest was exposed to a single irradiation with a total dose of 43 Gy. In addition, the irradiation field was 3x30 cm, the center dose point on the back of rats was 1 cm under the body surface and the irradiation range was 3 cm above the esophagus, while the rest of the rat was covered. ^60^Co therapy apparatus (SFCC-8000C type, SSD=80 cm, dose rate: 111 cGy/min, Shandong Xinhua Medical Instrument Co., Ltd., Shandong, China) was used for irradiation. On the 7th and 14th day after irradiation, the pathological changes of radiation-induced esophagitis were observed in the animal models.

### Grouping of experimental animals and administration method

A total of 128 Wistar rats were randomly divided into eight groups with 16 rats in each group, half male and half female. Group 1 (the normal group) was the blank control without any radiation or treatment. Group 2 (single radiation group 1) was sacrificed for evaluation on the 7th day after being irradiated with 43 Gy ^60^Co γ-rays. Group 3 (single irradiated group 2) was sacrificed for evaluation on the 14th day after being irradiated. Group 4 (prevention group 1) was treated with cWPROL at a normal dose of 0.475 g/ml, 2 ml 3 times a day and at an interval of 6 h from the 1st to 14th day after radiation for the prevention of radiation-induced esophagitis. Group 5 (prevention group 2) was treated with cWPROL at a high dose of 1.425 g/ml, 2 ml 3 times a day and at an interval of 6 h from the 1st to 14th day after radiation. Group 6 (treatment group 1) was treated with cWPROL at a normal dose of 0.475 g/ml, 2 ml 3 times a day and at an interval of 6 h from the 7th to 14th day after radiation for treatment of radiation-induced esophagitis. Group 7 (treatment group 2) was treated with cWPROL at a high dose of 1.425 g/ml, 2 ml 3 times a day and at an interval of 6 h from the 7th to 14th day after radiation. Group 8 (treatment group 3) was treated with a formulation of western medicines at a dose of 2 ml 3 times a day with an interval of 6 h from the 7th to 14th day after radiation. The rats in Groups 4, 5, 6, 7 and 8 were sacrificed for evaluation on the 14th day after being irradiated. The normal dose of cWPROL for the rats was converted from the human dose regulated according to the dosage standard of pharmacology (5,6). The formulation of the western medicine oral liquids was normal saline 250 ml, 2% lidocaine 20 ml, dexamethasone 10 mg and gentamicin sulfate 320,000 units. The concentration of oral western medicine for the rats was 0.16 times the human dose.

### Collection of blood samples

At each experimental time point, the experimental rats were anesthetized with 2% pentobarbital sodium via intraperitoneal injection (45 mg/kg), blood was harvested from the orbital sinus into EDTA-K_2_ anticoagulative tubes and mixed quickly for the analysis of T-lymphocyte subsets. An additional 3 ml of blood was collected from the femoral vein, centrifuged at a speed of 2,000 × g for 10min by Labofuge 400R centrifuge (Saimo Biotechonology development Corp. Ltd., Shanghai, China) and then the supernatant was collected and maintained at −80°C for evaluation of IgG and complement C3 in the serum.

### Immunofluorescence staining and flow cytometry

EDTA-K_2_ anticoagulative rat blood (100 *μ*l) was placed into the bottom of two tubes. FITC-labeled mouse anti-rat CD3 (10 *μ*l) and 10 *μ*l mouse anti-rat FITC-labeled CD4 and RPE-labeled CD8 double-labeled monoclonal antibodies was added to separate tubes. The components in the tubes were mixed, stained in the warm incubator in the dark for 30 min and then detected by flow cytometry (Epics XLII; Beckman Coulter, Miami, FL, USA). The automatic calibration program of the flow cytometry was run to refine its sensitivity, threshold, compensate fluorescence and photoelectric multiple voltage. Prior to testing, fluorescent microspheres of Flow-Check™ Fluorospheres (10 *μ*l) were employed as standard samples to regulate the coefficient of variability (CV) value of the instrument and control it within 2%. Following calibration, the percentages and absolute numbers of T lymphocyte subsets were analyzed by detecting immunofluorescence data with Expo 32 ADC software, and the percentages and absolute values of T lymphocytes (CD3^+^), Th cells (helper T lymphocytes, CD3^+^CD4^+^CD8^−^) and Tc cells (cytotoxic T lymphocytes, CD3^+^CD4^−^CD8^+^) and the Th/Tc ratio were determined.

### Immune nephelometry

Immune nephelometry was applied as previously described (7) to evaluate the levels of IgG and complement C3.

### Statistical analysis

The data were analyzed using the SPSS 13.0 software package (SPSS, Inc., Chicago, IL, USA). The comparisons between the different groups were made by one-way ANOVA. The Student-Newman-Keuls test was adopted when the variance was equal and the Kruskal-Wallis one-way analysis of variance H test was employed when the variance was unequal. The results were presented as mean ± standard deviation. P<0.05 was considered to indicate a statistically significant result.

## Results

### Evaluation of the white blood cells in the peripheral blood of the rats

**Figure 1.**
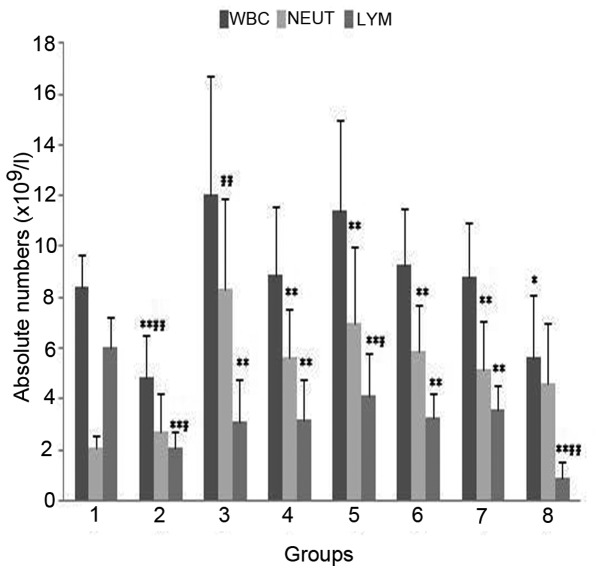
Absolute numbers of total white blood cells (WBC), neutrophile granulocytes (NEUT) and lymphocytes (LYM) in the peripheral blood of rats in each group. ^*^P<0.05 vs. Group 1, ^**^P<0.01 vs. Group 1, ^#^P<0.05 vs. Group 3, ^##^P<0.01 vs. Group 3.

**Figure 2.**
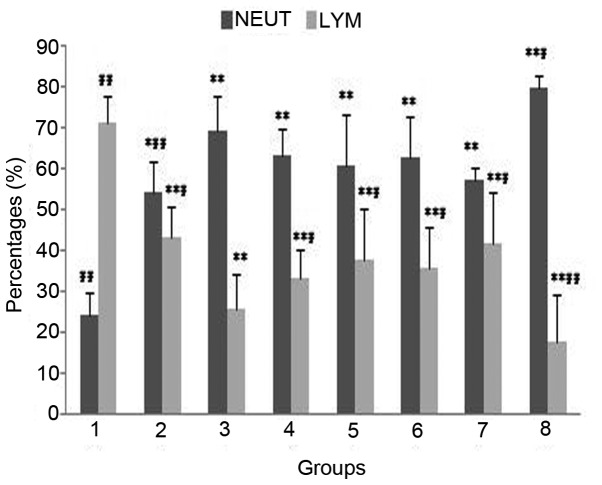
Percentages of neutrophile granulocytes (NEUT) and lymphocytes (LYM) in the peripheral blood of rats in each group. ^*^P<0.05 vs. Group 1, ^**^P<0.01 vs. Group 1, ^#^P<0.05 vs. Group 3, ^##^P<0.01 vs. Group 3.

The total number of white blood cells in the peripheral blood of the irradiated rats was significantly decreased compared with that of the normal group (Group 1), on the 7th day after irradiation (Group 2; P<0.001), but was not markedly different on the 14th day after radiation (Group 3; P>0.05; Fig. 1). The total numbers of white blood cells in the peripheral blood were within the normal range in the prevention and cWPROL treatment groups (Groups 4, 5, 6 and 7; P>0.05), but significantly lower than normal in the western medicine treatment group (Group 8; P<0.05; Fig. 1). The percentage of neutrophile granulocytes (NEUT) on the 7th day after irradiation (Group 2) was significantly elevated compared with that in the normal group (Group 1; P<0.05), furthermore, it was much higher on the 14th day after radiation (Group 3) than that in Group 2 (P<0.01; Fig. 2).

The absolute numbers and percentages of lymphocytes (LYM) in the peripheral blood of the irradiated rats (Groups 2, 3, 4, 5, 6, 7 and 8) were significantly lower than those in Group 1 (P<0.01; Figs. 1 and 2) and the percentage of LYM reached the minimum on the 14th day after radiation (Fig. 2). Compared with single radiation group 2 (Group 3), the percentage of NEUT in the western medicine treatment group (Group 8) was significantly increased (P<0.05) while the percentage and absolute number of LYM were significantly decreased in Group 8 (P<0.01; Figs. 1 and 2). In addition, the percentages of LYM in the groups treated with cWPROL (Groups 4, 5, 6 and 7) and the absolute number of LYM in the prevention group treated with a high dose of cWPROL (Group 5) were both higher compared with those of single radiation group 2 (Group 3; P<0.05; Figs. 1 and 2).

### Impacts on the quantity of T-lymphocyte subsets in each group

**Figure 3.**
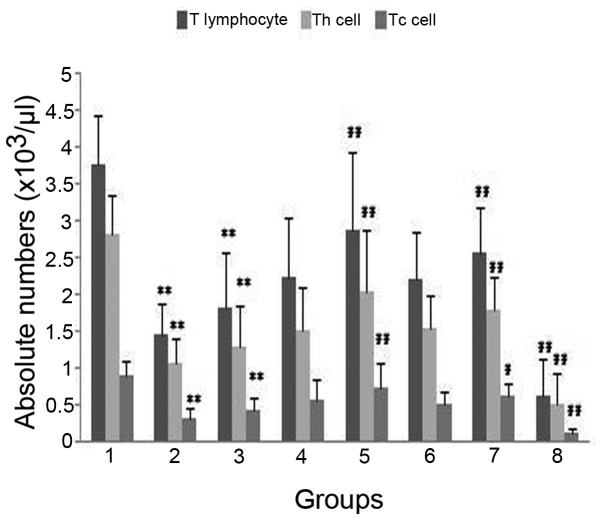
Absolute numbers of T lymphocytes (CD3^+^), Th cells (helper T lymphocytes, CD3^+^CD4^+^CD8^−^) and Tc cells (cytotoxic T lymphocytes, CD3^+^CD4^−^CD8^+^) in the peripheral blood of rats in each group. ^**^P<0.01 vs. Group 1, ^#^P<0.05 vs. Group 3, ^##^P<0.01 vs. Group 3.

**Figure 4.**
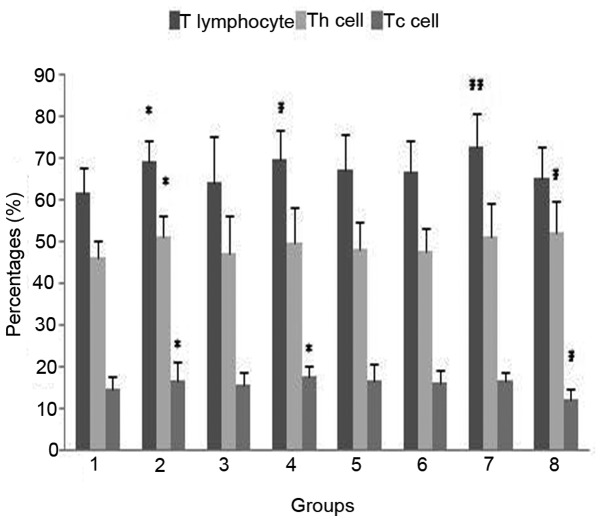
Percentages of T lymphocytes (CD3^+^), Th cells (helper T lymphocytes, CD3^+^CD4^+^CD8^−^) and Tc cells (cytotoxic T lymphocytes, CD3^+^CD4^−^CD8^+^) in the peripheral blood of rats in each group. ^*^P<0.05 vs. Group 1, ^#^P<0.05 vs. Group 3, ^##^P<0.01 vs. Group 3.

**Figure 5.**
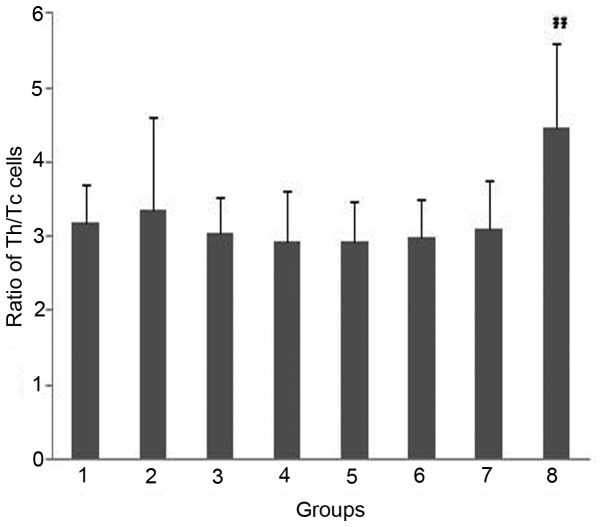
Ratios of Th cells (helper T lymphocytes, CD3^+^CD4^+^CD8^−^)/Tc cells (cytotoxic T lymphocytes, CD3^+^CD4^−^CD8^+^) in the peripheral blood of rats in each group. ##P<0.01 vs. Group 3.

In the single radiation groups (Groups 2 and 3), the absolute numbers of T lymphocytes, Th cells and Tc cells were markedly decreased on the 7th and 14th day after irradiation, (P<0.01; Fig. 3). The percentages of T lymphocytes, Th cells and Tc cells demonstrated a transitory rise on the 7th day (Group 2; P<0.05) and then returned to normal on the 14th day (Group 3; P>0.05) in comparison with Group 1 Fig. 4). There was no evident difference in the Th/Tc ratio between Groups 2, 3 and 1 (P>0.05; Fig. 5).

In the prevention group treated with a normal dose of cWPROL (Group 4), the absolute numbers of T lymphocytes, Th cells and Tc cells were not significantly different from those in Group 3 (P>0.05; Fig. 3). However, the percentage of T lymphocytes was higher than that in Group 3 (P<0.05; Fig. 4). A distinct elevation of the percentage of Tc cells was observed compared with that in Group 1 (P<0.05; Fig. 4). In the prevention group treated with a high dose of cWPROL (Group 5), the absolute numbers of T lymphocytes, Th cells and Tc cells were all distinctly greater than those of Group 3 (P<0.001; Fig. 3).

In the treatment group treated with a high dose of cWPROL (Group 7), the quantity of T lymphocytes rose significantly (P<0.01) after 8 days of treatment (14th day after radiation) and the absolute numbers of Th cells and Tc cells were markedly higher (P<0.05) compared with those in Group 3 (Fig. 3).

However, the absolute numbers of T lymphocytes, Th cells and Tc cells in the western medicine treatment group (Group 8) were significantly lower than those in Group 3 (P<0.001; Fig. 3), and the percentage of Th cells was markedly higher (P<0.05; Fig. 4); thus the ratio of Th/Tc increased significantly (P<0.01; Fig. 5).

### Analysis of the levels of IgG and complement C3 in the serum of rats in each group

**Figure 6.**
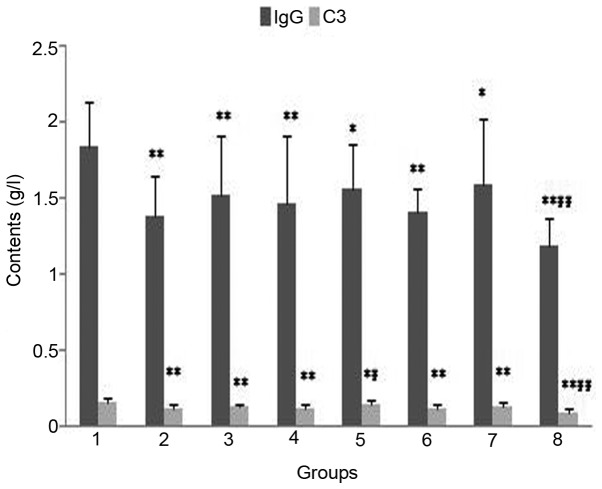
Contents of IgG and complement C3 in the serum of the rats in each group. *P<0.05 vs. Group 1, **P<0.01 vs. Group 1, #P<0.05 vs. Group 3, ##P<0.01 vs. Group 3.

Compared with the levels in Group 1, the levels of IgG and complement C3 in the serum were significantly decreased on the 7th and 14th day after radiation (Groups 2 and 3; P<0.01; Fig. 6). The level of IgG did not recover in the prevention and treatment groups treated with cWPROL (Groups 4, 5, 6 and 7; Fig. 6). The level of complement C3 in the prevention group treated with a high dose of cWPROL (Group 5) increased significantly (P<0.05) compared with that in Group 3, however it did not return to the normal level (Fig. 6).

## Discussion

Deficiency of cellular immunity and disordered humoral immunity have often been observed in patients with malignant tumors (8). Although radiotherapy is an effective method for preventing the growth of tumors, it further inhibits immunological function (1). In the current study, the percentage of lymphocytes in the peripheral blood of the Wistar rats was reduced significantly on the 14th day following irradiation with 43 Gy ^60^Co γ-rays. Consistent with the study by Kajioka *et al* (9), the absolute numbers of T lymphocytes, Th cells and Tc cells of the radiation-induced rats in the present study were significantly lower than those in the normal rats (P<0.001). The main cause of the phenomenon may be the downregulation of CD28 in T lymphocytes, which may lead to a high level of Fas-mediated apoptosis and therefore impair the immune system (10). T lymphocytes, particularly CD4^+^ and CD8^+^ T lymphocytes, play a leading role in the anti-tumor function *in vivo*; therefore, the quantities of CD4^+^ and CD8^+^ T lymphocytes are crucial for killing the cancer cell. T lymphocytes are easily injured by radiation due to their sensitivity to irradiation. The present study revealed that the immunological function of the rats with radiation-induced esophagitis was significantly impaired. Furthermore, the absolute numbers of T lymphocytes, Th cells and Tc cells of the rats in the prevention group treated with the high dose of cWPROL and in the cWPROL treatment groups were markedly elevated compared with those in the single radiation group 2 (Group 3), and the percentage of T lymphocytes was clearly increased in the prevention group treated with a normal dose of cWPROL. These results indicate a dose-effect relationship of cWPROL in the prevention and treatment of radiation-induced esophagitis. Furthermore, the immune repairing effect of cWPROL may aid the quick recovery of radiation-induced esophagitis and enhance the resistance of the body to tumors.

Immune globulins are the key molecules in humoral immunity, among which the majority are IgG, an important indicator that reflects the level of immunity of the body (11). Complement C3 is one type of globulin that has enzymatic activity and is able to kill tumor cells independently in nonspecific immunity and assist antibodies and immune cells to kill the cancer cells (12). Pathological changes such as exuviation, inflammatory cell infiltration and reduced levels of IgG and complement C3 occur in the radiation-induced rats, which results in bacterial infection (13). The current study showed that the levels of IgG and complement C3 significantly declined in irradiated rats. The levels of IgG in the prevention and treatment groups treated with cWPROL were not markedly different from that in the single radiation group 2 (Group 3). The level of complement C3 in the prevention group with a high dose of cWPROL was clearly recovered. These results suggest that cWPROL may weaken the pathogenicity of conditioned pathogens on the esophagus by strengthening the humoral immunity of the body.

Glucocorticoids, antibiotics and mucosal anesthetics are common medicines used in clinic to treat radiation-induced esophagitis and relieve pain by antibiosis, controlling secondary infection of local mucosa, alleviating exudation and edema, or paralyzing the sensory nerve endings in the esophageal mucosa. In addition, the improvement rate of combined therapy with these medicines is 72% (14). However, the present study demonstrated that the total numbers of white blood cells and lymphocytes, and the percentage of lymphocytes in the peripheral blood of irradiated rats decreased in the group treated with western medicine. Despite inhibiting the number and function of immune cells and chemotaxis of inflammatory cells, western medicine is able to control the inflammatory response and thus promote tissue repair, but its long-term use may harm the ability of the immune system to act against tumors. The rationality of preventive application of antibiotics on patients who receive radiotherapy remains to be discussed.

To summarize, cWPROL is extracted from a traditional Chinese herbal formulation that contains various Chinese herbal medicines and is highly effective in preventing and treating radiation-induced esophagitis in clinic. In this study, we discussed the likely functional mechanisms of cWPROL and revealed that by elevating the total number and percentage of lymphocytes, the absolute levels of T lymphocytes, Th cells and Tc cells, and the level of complement C3, cWPROL may repair the impaired immune system of rats with radiation-induced esophagitis, and thus postpone or treat the disease. Therefore, cWPROL not only prevents and treats complications of radiotherapy, but also enhances the cellular and humoral immunity of the body, thus improving the efficacy of anti-tumor activity.

## Abbreviations:

cWPROL: composition isolated from white peony root oral liquid
